# Rheological Study of Gelation and Crosslinking in Chemical Modified Polyamide 12 Using a Multiwave Technique

**DOI:** 10.3390/polym12040855

**Published:** 2020-04-07

**Authors:** Dominik Dörr, Ute Kuhn, Volker Altstädt

**Affiliations:** 1 Department of Polymer Engineering, University of Bayreuth, Universitätsstrasse 30, 95447 Bayreuth, Germany; dominik.doerr@uni-bayreuth.de (D.D.); ute.kuhn@uni-bayreuth.de (U.K.); 2 Bavarian Polymer Institute, Universitätsstrasse 30, 95447 Bayreuth, Germany

**Keywords:** rheology, multiwave, gelation, crosslinking, polyamide, PA12, chemical modification, chain extender, Joncryl, Vestamid

## Abstract

When processing particular polymers, it may be necessary to increase the molecular weight, for example, during polymer recycling or foaming. Chemical additives such as chain extenders (CE) are often used to build up the molecular weight during reactive extrusion. One issue of chain extenders, however, is that they can cause gelation or crosslinking of the polymer during processes with long residence times. This can lead to strong process fluctuations, undesired process shutdowns due to uncontrollable torque and pressure fluctuations and finally consistent material quality cannot be guaranteed. To measure and understand the reactivity between the polymer and the CE a rheological test can help. However, the standard gel point evaluation used for thermosets by examining the point of intersection of storage- and loss modules is not suitable, as this method is frequency-dependent. This study uses a multiwave rheology test to identify the gel-point more reliably. Both evaluation methods were compared on a polyamide 12 system, which is modified with an industrially relevant chain extender. The results show that the multiwave test can be applied on a chemical modified thermoplastic system and that the material system indicates a general tendency to crosslink. The frequency-independent gel-point evaluation shows that the gel-point itself is dependent on the processing temperature. Finally, it was possible to detect undesired side reactions, which are not recognizable with the standard testing method. Both findings are directly relevant for the reactive extrusion process and help to understand the mechanism of gelation.

## 1. Introduction

Chain extenders are widely used in the polymer industry, especially in the recycling industry, due to the chain degradation during processing at high temperatures and the resulting loss of properties [1]. Furthermore, they are often used in polymer foaming processes to increase the melt-strength of polyesters and polyamides to enhance the foamability and moldability of bead foams [2,3,4]. During chain extension a bi- or multifunctional reactant reacts with the end groups of polycondensates and the molecular weight increases [5]. There are three ways of chain extension: linear chain extension, non-linear chain extension and chain extension with polymeric chain extenders, where the last two are rather a multiple branching of the polymer chain [6]. Linear chain extension is rather ineffective since only two chains are bonded together and the molecular weight only increases slightly (doubles) in comparison to other methods. The other two ways are based on a multifunctional branching reaction and the molecular weight is increased with the number of functional groups of the additive [6]. Nevertheless, all methods are described as chain extensions in the literature [5,7,8,9].

**Figure 1:**
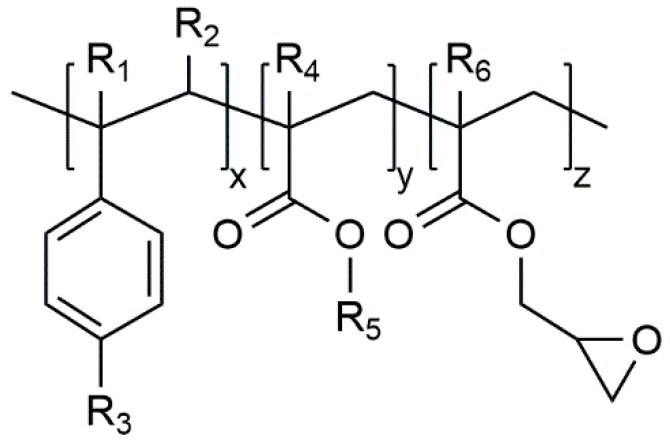
General structure of styrene–acrylic multi-functional chain extenders according [1].

**Figure 2:**
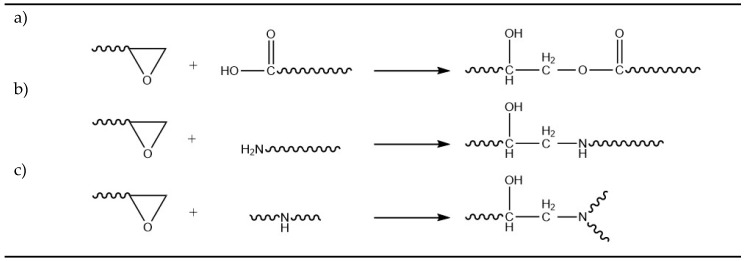
Possible reactions of the epoxy group and polyamide 12. Reaction between (**a**) epoxy- and carboxyl group and (**b**,**c**) epoxy- and amine groups.

Polymeric chain extenders are mostly multi-functional styrene-acrylic based polymers. Their general chemical structure is shown in Figure 1 [5]:

According to Villalobos et al. [1] the organic moieties R_1_ to R_5_ are H, CH_3_, a higher alkyl group or their combinations, R_6_ is an alkyl group and *x*, *y*, and *z* are each between 1 and 20, thus oligomeric extenders are possible. The functionality, in this case the number of reactive epoxy groups, is higher than four (*f*_n_ > 4). These epoxy groups react with functional groups of the polymer and consequently a long chain branched material can be achieved. The possible reactions of a multi-functional epoxy and polyamide are with the carboxyl group (cf. Figure 2a), amine groups (cf. Figure 2b,c), and imide group (after coupling reaction between amine and epoxy) [10]. By using a higher amount of the chain extender, the chain extender crosslinks the polymer. [10,11].

Frequently a change in the polymer topology, caused either by a branching or crosslinking reaction, directly influences the rheological behavior of the polymer melt. The point of crosslinking of modified thermoplastics is comparable with the point of gelation/gel-point of curing systems like thermosets and represents the liquid-solid transition, which is caused by the formation of a network [12,13,14]. In contrast to thermoset systems, the gelation of a reactive modified thermoplastic system is not desired, since inhomogeneities and process fluctuations can occur. Therefore, a later occurring gel-point is beneficial for thermoplastic systems. To determine this gel-point, oscillatory shear rheology is used commonly. During the reaction the storage modulus (*G*′) increases, while the loss modulus (*G*″) only rises slightly, caused by an increase in molecular weight [12,15]. The cross-over point of the loss and storage modulus may represent the liquid-solid transition and thus the point of crosslinking/point of gelation [12,13]:

(1) $$G^{\prime }=G^{\prime \prime }or\;\frac{G^{\prime \prime }}{G^{\prime }}=\tan\left(\delta \right)=1$$

However, as Winter [16] stated in the late 1980s, the stress relaxation at gel-point shows a power law behavior:

(2) $$G\left(t\right)=St^{-n}$$

with the gel strength *S* and the relaxation exponent n (0 < *n* < 1) [16]. From Equation (2) the individual equations of storage and loss modulus at gel point can deducted by using the wave function [12,14,16,17]:

(3a) $$G^{\prime }\left(\omega \right)=n!\cos\left(\frac{n\cdot \pi }{2}\right)S\omega^{n}$$

(3b) $$G^{\prime \prime }\left(\omega \right)=n!\sin\left(\frac{n\cdot \pi }{2}\right)S\omega^{n}$$

Thus, the detection of the gel-point by the cross-over of *G*′ and *G*″ is only valid for a limited kind of polymers and matching conditions, which lead to a relaxation exponent of 0.5:

(4) $$Crossover=G^{\prime }\left(T,\;\omega \right)=G^{\prime \prime }\left(T,\;\omega \right)=\sqrt{\frac{\pi }{2}}S\left(T\right)\omega^{0.5}$$

This is valid for example for polymers with a stoichiometric balance, an excess of cross-linker and only at temperatures much above glass transition temperature (*T*_g_), because the *T*_g_ can be shifted by changing the heating rate [16]. Otherwise the cross-over point of *G*′ and *G*″ does not represent the liquid-solid transition and gives only a rough estimation. In general, the necessary conditions are never met.

Hence, by changing the frequency of the measurement, the gel-point can be shifted to shorter or longer times. Only the loss factor (tan(*δ*)) is frequency independent by using Equations (3a) and (3b) [14,16]:

(5) $$\tan\left(\delta \right)=\frac{G^{\prime \prime }}{G^{\prime }}=\tan\left(\frac{n\cdot \pi }{2}\right)$$

**Figure 3:**
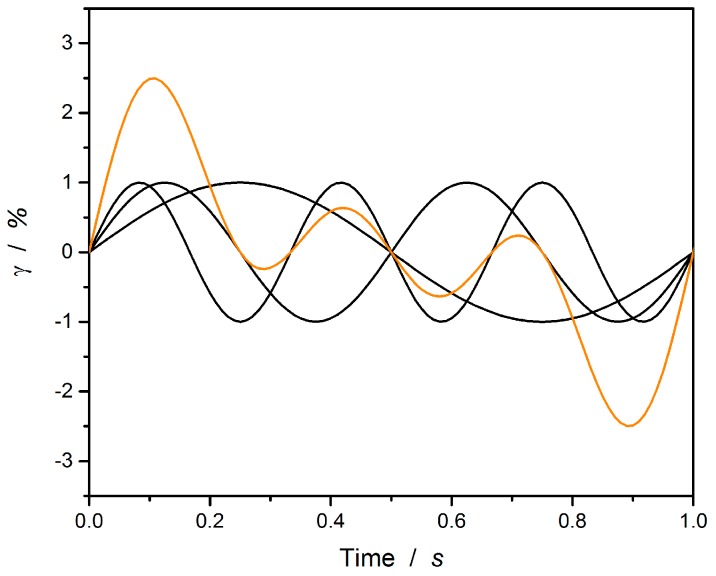
Superposition (orange) of three individual frequencies (black).

Malkin et al. [18,19] proposed an experimental method for determining the dynamic characteristics of polymers by using a computer-based Fourier Transform mechanical spectroscopy technique. Different frequencies (cf. Figure 3, black lines) are superimposed on a strain function (cf. Figure 3, orange line). The sample is exposed to this strain function and the resulting stress and torque are measured [20]. This principle was adapted by Winter and Holly [12,20] for detecting the gel-point, by investigation of the loss factor.

It has to be guaranteed, that the resulting amplitude *γ_i_* is still in the linear viscoelastic range *γ_c_* [20]:

(6) $$\overset{n}{\underset{i=1}{\sum }}\gamma_{i}\leq \gamma_{c}$$

The resulting stress signal is separated to the individual stress signals by a discrete Fourier transformation [18,19,20]. Nowadays this method is known as a “multiwave test” [13] and can be run and automatically evaluated by the rheometer software, whereby the basic principle is further optimized [21].

All investigations using the multiwave test are based on low molecular weight monomers, mainly in the field of curing systems, like polyurethanes [19] or epoxy resins [22]. Some other publications exist about UV-curable thiolene systems [23,24] or acrylic-based polymers [25]. To the authors’ best knowledge, there is no publication about multiwave tests on thermoplastic reactive systems. One publication was found using the multiwave technique for investigations regarding the recrystallization of polypropylene [26]. Although the gelation of thermoplastic systems during processing is undesirable, there is currently no understanding of the mechanisms involved. During polymer foaming processes with long residence times (for homogenization and cooling), particular gelation can occur, which (temporarily) blocks the nozzles and reduces the quality of the product. This study shows that the multiwave test method can be applied on chemical reactive thermoplastics and the results giving much more information than the conventional evaluation method with the crossover of storage and loss modulus, such as about side reactions. Therefore, polyamide 12 is modified with a commercial chain extender (CE) in different concentrations and analyzed with the multiwave technique. Polyamide 12 was chosen because of the many possible reactions with the chain extender (cf. Figure 2) and at the same time a low tendency for self-reaction and molecular decomposition.

## 2. Materials and Methods

This work is based on commercially available materials. Polyamide 12 is a grade called Vestamid LX9012 produced by EVONIK Industries AG (Essen, Germany). The polymer was cryo-milled and afterwards vacuum dried at 80 °C for 16 h prior use. The chain extender is a multifunctional epoxy oligomer (Joncryl 4468) provided by BASF SE (Ludwigshafen, Germany).

The geometry of the samples used for the measurements was 25 mm in diameter and 1.25 mm in height. These samples were fabricated with a PW10 hydraulic press, made by Paul-Otto Weber GmbH (Remshalden, Germany). The milled pellets were mixed with a milled chain extender (1 and 5 wt %), mixed and filled into a mold, which was exposed to a temperature of 200 °C. After 2 min a stamping force of 50 kN was applied for 2 min. Afterwards the mold was fast cooled in a water-cooled press with an initial pressure force of 50 kN. This preparation should prevent reaction between CE and polymer. The prepared samples were stored in a desiccator and measured within the next 24 h.

Oscillatory tests were done with a MCR 702 instrument (Anton Paar, Graz, Austria) and plate-plate geometry with a 1 mm gap. All samples were handled the same way and a fixed time of 5 min for heating was applied before the measurement was started. First, the linear viscoelastic (LVE) range of the polymer was investigated with an amplitude sweep with a constant angular frequency (*ω* = 1 rad/s) and a logarithmic amplitude ramp from 0.01% to 100% at 200 °C. Multiwave tests were done to investigate the state of reaction. The fundament frequency was *ω*_0_ = 1 rad/s with an amplitude of 3%. The other frequencies are multiples of the fundamental frequency, *ω*_1_ = 5 rad/s and *ω*_2_ = 25 rad/s, each with an amplitude of 3%. The amplitude factor was 3, which gave a resulting max. amplitude of 9%. The used temperatures were 200, 230, 240, 250 and 260 °C, respectively.

## 3. Results and Discussion

**Figure 4:**
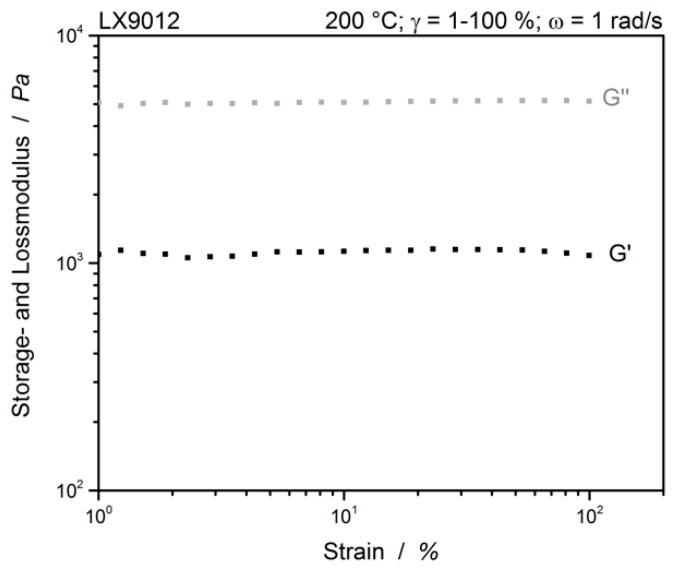
Amplitude sweep of LX9012 without chain extender measured at 200 °C with 1 rad/s.

During the implementation of the multiwave tests, the time-dependent strains of the individual waves are summed up. Therefore, it must be ensured in the tests that the LVE is met at all times (Equation (5)). Figure 4 shows the amplitude sweep of neat LX9012.

**Figure 5:**
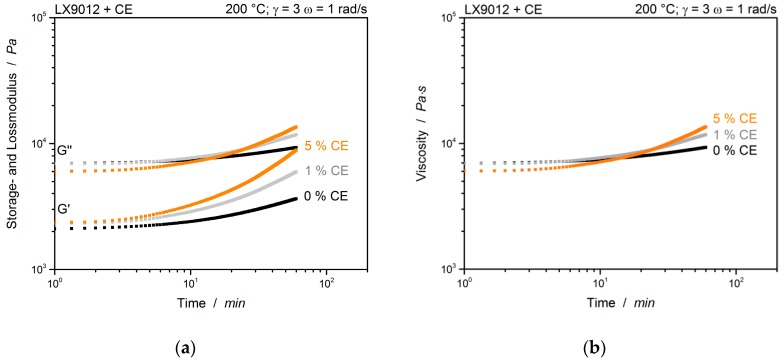
Time sweep of unmodified and chain extender modified LX9012 measured at 200 °C with only 1 rad/s. (**a**) Storage- and loss modulus; (**b**) Viscosity over time.

The strain sweep of LX9012 shows the characteristics of a viscoelastic liquid, where the viscous behavior dominates: $G^{\prime \prime }>G^{\prime }$. The LVE is between 2% and 20%. For all following measurements a single curve strain of *γ*_i_ = 3% is chosen, resulting in a maximum strain of 9% during the multiwave tests, which is still in the LVE. Figure 5 shows a standard time sweep with constant temperature of 200 °C, constant amplitude (3%) and only one constant frequency of all prepared materials without and with chain extender.

The moduli as well as the viscosity have approximately the same initial value. It can be assumed, therefore that the reaction time can be neglected during preparation, hence it is the same time for each sample, and the reaction can be observed during the measurement in the rheometer. The slightly lower viscosity of the sample with 5% CE at start up, can be attributed to the chain extender itself, which acts like a plasticizer. Besides the two modified samples, the unmodified sample (0% CE) shows an increase in viscosity after about 10 min, too. According to Dijkstra et al. [27] this increase is caused by polycondensation reactions between carboxyl end groups and amine end groups of the polymer and consequently an increase in molecular weight. This self-reaction behavior of LX9012 is less pronounced than the reaction between the polymer and the chain extender and can be neglected for the measurements. Hence no cross-over point of *G*′ and *G*″ can be observed at 200 °C, the temperature is increased to 240 °C, where a higher reactivity of the CE is expected.

**Figure 6:**
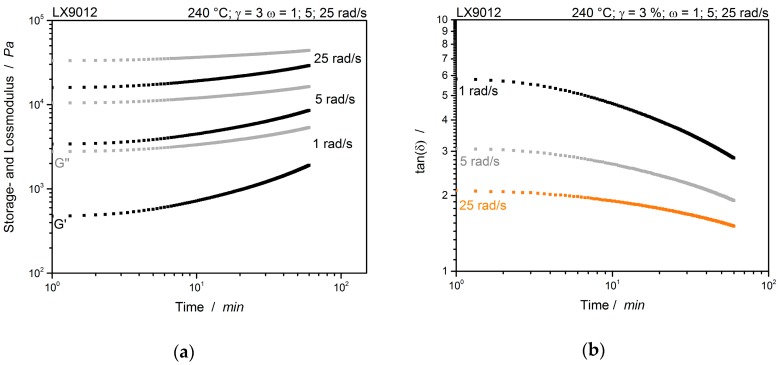
Multiwave test of unmodified LX9012 as reference measured simultaneous with different frequencies. (**a**) Storage- and loss modulus and (**b**) tan(*δ*). Measured at 240 °C.

For the determination of crosslinking or even the branching behavior of the PA12, initial multiwave tests are conducted. This should give an insight into the multiwave behavior of a non-reactive system, which is shown in Figure 6.

**Figure 7:**
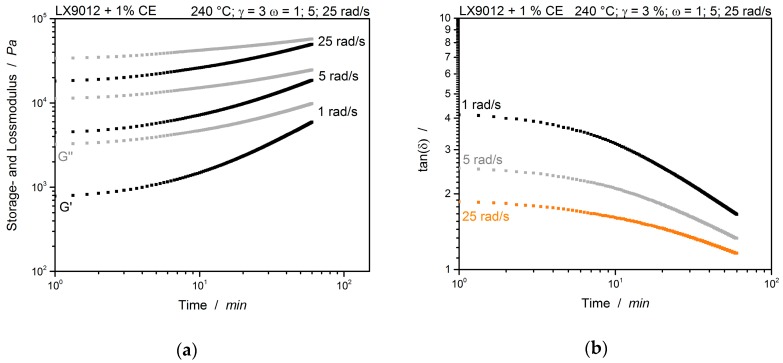
Multiwave test of LX9012 modified with 1% CE measured simultaneous with different frequencies. (**a**) Storage- and loss modulus and (**b**) tan(*δ*). Measured at 240 °C.

The typical polymer time-temperature shift (TTS) can be seen in Figure 6. Increasing velocity (frequency) and temperature have a comparable effect on a sample’s rheological behavior. Testing with higher frequencies has the same effect than cooling down the sample. Both resulting in higher storage and loss moduli [13]. As shown previously, the moduli slightly increase after about 10 min in the unmodified sample. Either a crossover of the moduli nor a frequency independent cross-point of the tan(*δ*) are notable. This observation is transferable to the PA12 modified with 1% CE (cf. Figure 7).

**Figure 8:**
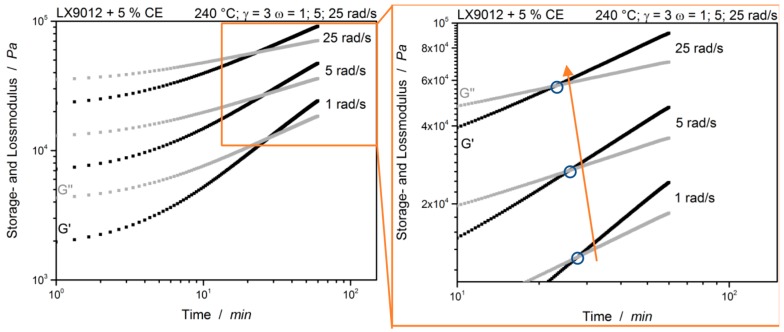
Time Sweeps of LX9012 with 5% chain extender measured at 240 °C with multiwave test. Gel-point is highlighted.

Here only branching occurs and no gel-point is detectable within the tested time frame of 60 min. This supports the specific feature of the chain extender: A broaden and gel-free processing window [1]. After proving the non-reactivity of the unmodified polymer and the gel-free reactivity with a low CE concentration, a sample with 5% CE is investigated. First, the cross-over point of the moduli is evaluated. This is illustrated in Figure 8.

It is noticeable that the cross-over point of the two moduli with higher frequency is moved to lower times. The point is shifted from about 27.8 min at 1 rad/s to 22.8 min at 25 rad/s. The observation already made by Winter [16] that the cross-over point of the two moduli only describes the gel-point under certain requirements (see above) can now also be transferred to reactive thermoplastic systems or chemical modified systems. The main reason for the frequency dependent shift could be the non-stoichiometric balance between the CE and the polymer, hence no excess of CE is used.

**Figure 9:**
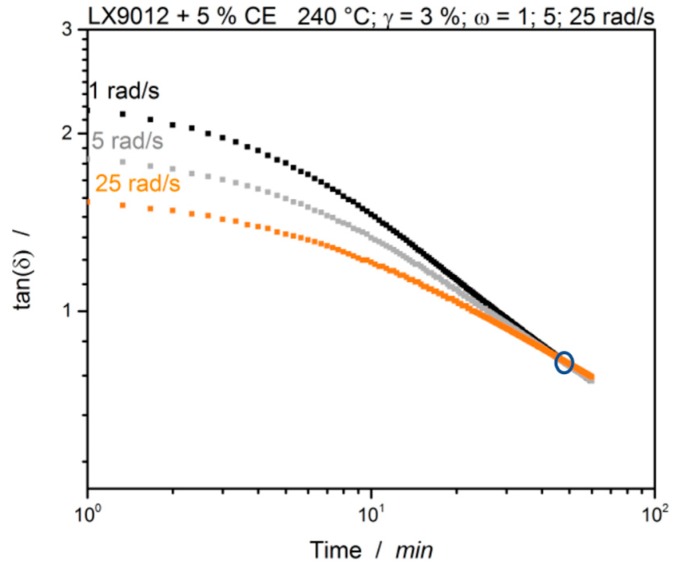
LX9012 with 5% CE measured at 240 °C. tan(*δ*) of different simultaneous measured frequencies for the evaluation of the gel-point (highlighted).

Figure 9 shows the suggested evaluation according to Winter et al. with the frequency independent loss factor [14,16].

The three simultaneous measured loss factor curves coincide a single point. This point is frequency independent and represents the gel-point of the sample. Compared to the previously mentioned cross-over point of *G*′ and *G*″, the gel-point is located at much later times. In this case it is at about 46.2 min. The observation is consistent with Winter’s theoretical assumptions that a system with tendency to crosslink leads to an exponent value of n > 0.5 and finally shows power law behavior [16]. The used system with 5% CE can be assumed to be sub stoichiometric. This indicates a noticeably low tendency of the material to crosslink than expected with the standard evaluation. It can be assumed therefore that not the whole material crosslinks, but particular gelation can occur. During polymer processing, this tendency can be increased by higher shear rates and local temperature hot spots. Although the individual points indicate that this is the gel point because the system shows power law behavior, there is only one intersection of the curves and the material was rubbery after the measurement, only NMR measurements can finally provide information about the molecular structure.

**Figure 10:**
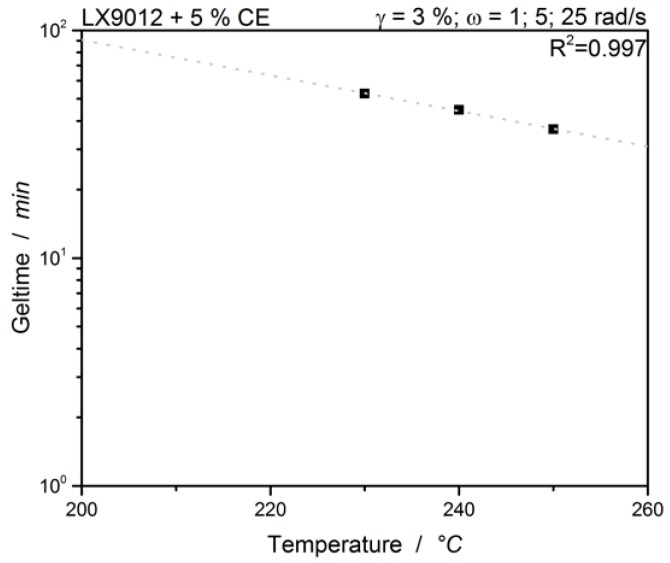
Semi-logarithmic plot of gel-time vs. temperature of LX9012 with 5% CE.

Besides the standard evaluation of the gel-point, the multiwave tests give much more information on the sample reactivity. By plotting the evaluated gel-time tested at different temperatures in a semi-logarithmic scale it is possible to investigate the gel-point temperature dependency (cf. Figure 10).

**Figure 11:**
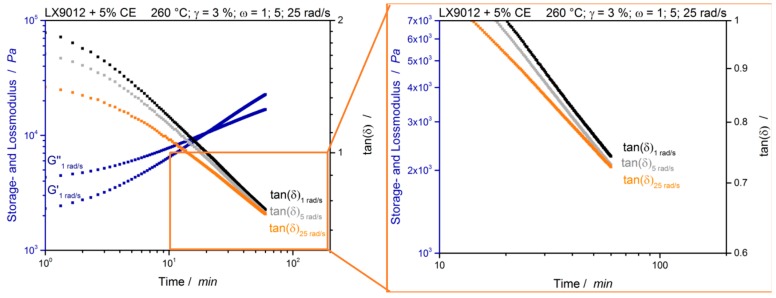
LX9012 with 5% CE measured at 260 °C. tan(*δ*) of different simultaneous measured frequencies for the evaluation of the gel-point.

First, it is shown, that the evaluation of the gel point with multiwave tests can be applied at different temperatures. The results show that the gel point depends on the test temperature. At low temperatures (230 °C) the gel point occurs after about 52 min whereas a network is built at higher temperatures (250 °C) after about 37 min. In addition, the reaction kinetic can be determined by this plot. Here an Arrhenius like behavior can be observed, which allows to estimate the gel-point at higher and lower temperatures, that may be more process relevant. The results of a measurement at a higher temperature of 260 °C are shown in Figure 11.

The cross-over point of the storage and loss modulus indicates a cross over point at about 20 min. In contrast the loss factors of the curve with 1 rad/s and 5 rad/s are approaching each other in the first 10 min, but after about 20 min they run parallel to each other and do not intersect each other after 60 min (cf. Figure 11, magnification). This indicates, that at the tested temperature no gel-point is achieved. This could be related to secondary reactions during the measurement. Either a degradation of the PA12, an undefined self-reaction of the CE [28] or both together could occur at the tested temperature and prevent the formation of a network.

## 4. Conclusions

A study on a chemically modified PA12 was performed to understand the possibility of using the multiwave test to show the possibility of gelation and crosslinking of a reactive thermoplastic under applied shear and thermal load. The cross-over point of storage and loss modulus is frequency dependent and therefore less suitable to detect the gel-point in thermoplastic systems. Next a multiwave test was applied on the PA12 and CE system and a frequency independent gel-point was detected. In comparison to the classical procedure, this point is shifted to much longer times indicating a low tendency of the material to crosslink, even at lower chain extender concentrations. Finally, it was shown that the multiwave technique is able to detect side reactions resulting from degradation during the measurements, when no cross-over point of the different loss factors is achieved.
